# Genome-wide Screens for Sensitivity to Ionizing Radiation Identify the Fission Yeast Nonhomologous End Joining Factor Xrc4

**DOI:** 10.1534/g3.114.011841

**Published:** 2014-05-21

**Authors:** Jun Li, Yang Yu, Fang Suo, Ling-Ling Sun, Dan Zhao, Li-Lin Du

**Affiliations:** National Institute of Biological Sciences, Beijing 102206, China

**Keywords:** nonhomologous end joining, *Schizosaccharomyces pombe*, XRCC4

## Abstract

Nonhomologous end joining (NHEJ) is the main means for repairing DNA double-strand breaks (DSBs) in human cells. Molecular understanding of NHEJ has benefited from analyses in the budding yeast *Saccharomyces cerevisiae* and the fission yeast *Schizosaccharomyces pombe*. In human cells, the DNA ligation reaction of the classical NHEJ pathway is carried out by a protein complex composed of DNA ligase IV (LigIV) and XRCC4. In *S. cerevisiae*, this reaction is catalyzed by a homologous complex composed of Dnl4 and Lif1. Intriguingly, no homolog of XRCC4 has been found in *S. pombe*, raising the possibility that such a factor may not always be required for classical NHEJ. Here, through screening the ionizing radiation (IR) sensitivity phenotype of a genome-wide fission yeast deletion collection in both the vegetative growth state and the spore state, we identify Xrc4, a highly divergent homolog of human XRCC4. Like other fission yeast NHEJ factors, Xrc4 is critically important for IR resistance of spores, in which no homologous recombination templates are available. Using both extrachromosomal and chromosomal DSB repair assays, we show that Xrc4 is essential for classical NHEJ. Exogenously expressed Xrc4 colocalizes with the LigIV homolog Lig4 at the chromatin region of the nucleus in a mutually dependent manner. Furthermore, like their human counterparts, Xrc4 and Lig4 interact with each other and this interaction requires the inter-BRCT linker and the second BRCT domain of Lig4. Our discovery of Xrc4 suggests that an XRCC4 family protein is universally required for classical NHEJ in eukaryotes.

In eukaryotic cells, DNA double-strand breaks (DSBs) can be repaired by two pathways, homologous recombination (HR) and nonhomologous end joining (NHEJ). HR uses an intact DNA duplex as the repair template, whereas NHEJ does not need a template and can join two DNA ends in the absence of any base-pairing at the junction. NHEJ is the predominant pathway for DSB repair in mammalian cells. In organisms in which HR is the favored pathway, such as the budding yeast *Saccharomyces cerevisiae* and the fission yeast *Schizosaccharomyces pombe*, NHEJ usually plays a minor role in DSB repair but becomes important when homologous templates are unavailable.

The best understood NHEJ mechanism relies on a group of conserved proteins including the Ku heterodimer and a specialized DNA ligase called Ligase IV (LigIV) in humans (Lieber 2010; Davis and Chen 2013). DSB repair mediated by this Ku-dependent and LigIV-dependent mechanism has been referred to as the classical or canonical NHEJ to distinguish it from end joining repair occurring independently of Ku and LigIV (Deriano and Roth 2013; Chiruvella *et al.* 2013).

In classical NHEJ, LigIV catalyzes the ligation reaction to rejoin the DNA ends. In most organisms in which classical NHEJ has been characterized, the ligation function of LigIV needs two cofactors, which are called XRCC4 and XLF/Cernunnos in humans. XRCC4 binds the C-terminal region of LigIV, which contains two BRCT domains (Critchlow *et al.* 1997). These two proteins form a tight complex composed of one molecule of LigIV and two molecules of XRCC4 (Sibanda *et al.* 2001; Wu *et al.* 2009). Budding yeast orthologs of LigIV and XRCC4, called Dnl4 and Lif1, respectively, also interact with each other in a manner dependent on the C-terminal BRCT-containing region of Dnl4 (Herrmann *et al.* 1998; Doré *et al.* 2006).

In the fission yeast *S. pombe*, studies of classical NHEJ have revealed the essential roles of the Ku heterodimer Pku70-Pku80, the LigIV ortholog Lig4, and the XLF ortholog Xlf1 (Manolis *et al.* 2001; Decottignies 2005; Hentges *et al.* 2006; Cavero *et al.* 2007; Li *et al.* 2012). However, no ortholog of XRCC4 has been found in *S. pombe*. The lack of a detectable XRCC4 ortholog in *S. pombe* has led to the proposition that such a factor may not always be required for classical NHEJ (Hentges *et al.* 2006; Wilson 2007; Cavero *et al.* 2007).

Here, we report the identification of a distant sequence homolog of XRCC4 in *S. pombe* and present evidence that it is essential for classical NHEJ and performs roles similar to human XRCC4. Our findings suggest that XRCC4 is a universally required component of classical NHEJ.

## Materials and Methods

### Strains and plasmids

Fission yeast strains used in this study are listed in Supporting Information, Table S1. Plasmids used in this study are listed in Table S2. Genetic methods for strain construction and composition of media are as described (Forsburg and Rhind 2006). In DY4792, a *natMX* marker was introduced by PCR-based gene targeting so that it replaced the genomic DNA between coordinates 2127216 and 2127259 on chromosome 2 in the intergenic region between *SPBC23G7.14* and *rpp202*. The *xrc4* deletion strains were constructed by PCR amplifying the deletion cassette in the Bioneer deletion strain and transforming the PCR product into strains from our laboratory strain collection. For the construction of plasmids expressing fluorescent protein–tagged Xrc4 and Lig4, the coding sequences of these two proteins were amplified by PCR from genomic DNA and inserted into modified pDUAL vectors (Matsuyama *et al.* 2004), which contain the *P41nmt1* promoter and the sequence encoding GFP or mCherry. The plasmids were linearized with *Not*I and integrated at the *leu1* locus. To allow the integration and selection of a second pDUAL-based plasmid, the *leu1^+^* marker in the first integrated plasmid was disrupted by PCR-based gene targeting using a PCR template in which an SVEM-hphMX marker (Erler *et al.* 2006) was inserted into an EcoNI site in the coding sequence of *leu1^+^*.

### Ionizing radiation sensitivity screens

We constructed the deletion mutant pool using the Bioneer version 1.0 haploid library and the Bioneer version 1.0 upgrade package as described (Sun *et al.* 2013). For the screen of vegetatively growing cells, the mutant pool pre-grown in YES medium was treated with 500 Gy of ionizing radiation (IR) using a ^137^Cs Gammacell 1000 irradiator (dose rate 16 Gy/min), grown for five OD_600_ doublings in YES medium, and then harvested for genomic DNA preparation. In parallel, an untreated control culture was grown for five OD_600_ doublings and then harvested. For the screen of spores, we first mated the mutant pool with DY4792 on SPAS plates. The mating mixture was digested with glusulase and spores were purified using a Percoll gradient as described (Sun *et al.* 2013). Approximately 3×10^7^ spores were incubated in YES medium to allow germination to occur. After 22 hr, cells were diluted to OD_600_ ≈0.1 in YES medium containing 20 mg/liter of G418 and 10 mg/liter of clonNAT, grown to OD_600_ ≈1.2, and plated on YEPD plates at a sufficiently low density so that single-clone colonies could form. Iodine staining indicated that approximately 50% of the colonies contained spores, consistent with the expectation that half of the cross progenies may contain the *fus1* deletion. After incubating for 6 d, approximately 400,000 colonies were harvested from the YEPD plates. Glusulase digestion and spore purification were performed as above. Approximately 4×10^7^ spores were treated with 100 Gy of IR and then allowed to germinate and grow in YES medium. After 26 hr, cells were harvested. In parallel, untreated spores were germinated and grown in YES medium for 26 hr and then harvested. Genomic DNA extraction, barcode PCR, Illumina sequencing, and sequencing data analysis were performed as described (Sun *et al.* 2013). The sequencing data are publicly available at NCBI Sequence Read Archive (http://www.ncbi.nlm.nih.gov/sra/) under the accession number SRX475058. The data are composed of four runs. Run SRR1174920 corresponds to untreated sample of the vegetative screen (uptag index is CGAT and dntag index is TATA); Run SRR1174919 corresponds to IR-treated sample of the vegetative screen (uptag index is TAAT and dntag index is AGGA); Run SRR1174923 corresponds to untreated sample of the spore screen (uptag index is TGCA and dntag index is GTCA); and Run SRR1174921 corresponds to IR-treated sample of the spore screen (uptag index is CTGA and dntag index is GCTA). For gene set enrichment analysis (GSEA), the lists of genes ranked by log2(control/treatment) ratios were analyzed using the preranked tool of GSEA v2.0.13 (Subramanian *et al.* 2005). The GO-derived MSigDB format gene sets were downloaded from the GO2MSIG website (http://www.go2msig.org/cgi-bin/prebuilt.cgi). The high-quality annotations-only gene sets of September 2013 were used.

### Spore IR sensitivity assay

Strains of the *h^90^* mating type were spotted on SPAS plates to induce mating and sporulation. Mating mixtures were digested with glusulase overnight to eliminate nonspore cells. Spores were treated with IR at the indicated doses, plated on YES plates, and allowed to grow at 30° until the appearance of colonies.

### Extrachromosomal DSB repair assay

The efficiency of repairing an extrachromosomal DSB was determined using the *ura4^+^* circularization assay (Decottignies 2005). The *ura4^+^* gene was PCR-amplified using the pREP2 plasmid as template, 5′-TAGCTACAAATCCCACTGGC-3′ and 5′-TTGACGAAACTTTTTGACAT-3′ as primers, and KOD-Plus-Neo (TOYOBO) as polymerase. The PCR product was first digested with DpnI at 37° for 4 hr to eliminate the template DNA, and then purified using the Illustra GFX kit (GE Healthcare). Fission yeast strains lacking *ura4^+^* and *his3^+^* genes were transformed with a mixture of the *ura4^+^* PCR product and an episomal plasmid pLD160, which contains the *his3^+^* gene and serves as a transformation efficiency control. For each transformation, 225 ng of *ura4^+^* PCR product and 225 ng of pLD160 were used. Ura^+^ and His^+^ transformants were selected on minimal media lacking uracil or histidine, respectively. The *ura4^+^* circularization efficiency was calculated as the number of Ura^+^ transformant colonies divided by the number of His^+^ transformant colonies.

### Chromosomal DSB repair assay

The efficiency of repairing a chromosomal DSB was determined using the HO survivor assay (Li *et al.* 2012). Briefly, log-phase cells grown in EMM minimal medium supplemented with 1.5 μM of thiamine were washed with water twice and plated onto an EMM plate without thiamine (−T). As a control, cells were also plated onto an EMM plate containing thiamine (+T). The survival rate was calculated as the number of colonies formed on the −T plate divided by the number of colonies on the +T plates. To analyze the DSB repair junction patterns, approximately 2000 survivor colonies from the −T plates were harvested for each strain. Genomic DNA was extracted and the repair junctions were amplified by PCR using primers 5′-AATGATACGGCGACCACCGAGATCTACACTCTTTCCCTACACGACGCTCTTCCGATCTxxxgaattcggccaggtacct-3′ and 5′-CAAGCAGAAGACGGCATACGAcgcacgtcaagactgtca-3′ (uppercase letters are Illumina sequencing adaptor sequences, xxx is the multiplexing index sequence, and the other lowercase letters are the sequences annealing to the genomic DNA). For an intact HO site, a 242-bp PCR product is expected. The PCR products were gel-purified and sequenced using an Illumina HiSequation 2000 for 49 cycles. The sequencing data are publicly available at NCBI Sequence Read Archive (http://www.ncbi.nlm.nih.gov/sra/) under the accession number SRX481656. The data are composed of four runs. Run SRR1184202 corresponds to wild-type sample (index is ATG); Run SRR1184205 corresponds to *pku70* mutant sample (index is CGT); Run SRR1184204 corresponds to *lig4* mutant sample (index is GCA); and Run SRR1184203 corresponds to *xrc4* mutant sample (index is TAC). For data analysis, we extracted reads starting with the 21-nt sequence xxxgaattcggccaggtacct. After trimming off the 21-nt sequence, the reads from the same survivor pool were compared to each other and identical sequences were grouped together.

### Light microscopy

Cells were stained with 0.5 μg/ml Hoechst 33342 in water for 10 min and then washed once with water before imaging. Live cell imaging was performed using a DeltaVision PersonalDV system (Applied Precision) equipped with a CFP/YFP/mCherry filter set (Chroma 89006 set) and a Photometrics CoolSNAP HQ2 camera. Images were acquired with a 100×, 1.4-NA objective, and analyzed with the SoftWoRx software.

### Immunoprecipitation

Approximately 100 OD_600_ units of log-phase cells grown in thiamine-free EMM medium were lysed by glass bead beating in the lysis buffer (50 mM HEPES, pH 7.5, 1 mM EDTA, 150 mM NaCl, 10% glycerol, 1 mM PMSF, 1 mM DTT, 0.05% NP-40, 1× Roche protease inhibitor cocktail). GFP-trap agarose beads (ChromoTek) were used for immunoprecipitating the GFP-tagged protein. After washing the beads five times with lysis buffer, proteins bound to beads were eluted by boiling in SDS-PAGE loading buffer.

### Yeast two-hybrid analysis

For yeast two-hybrid analysis, we used the Matchmaker system (Clontech). The cDNA of the *xrc4* gene was cloned into a prey vector modified from the pGAD GH vector (Clontech). The cDNA of the *lig4* gene and fragments of it were cloned into a bait vector modified from the pGBKT7 vector (Clontech). Bait and prey plasmids were co-transformed into the AH109 strain and transformants were selected on the double dropout medium (SD/−Leu/−Trp). The activation of the *HIS3* and *ADE2* reporter genes was assessed on the quadruple dropout medium (SD/−Ade/−His/−Leu/−Trp).

## Results

### Genome-wide screens for IR-sensitive mutants in fission yeast

During vegetative growth, *S. pombe* cells spend most of their time in the G2/M phase of the cell cycle, and DSBs are predominantly repaired by homologous recombination using the sister chromatids as recombination templates. As a consequence, vegetatively growing *S. pombe* NHEJ mutants are no more sensitive to IR than the wild-type (Manolis *et al.* 2001; Ferreira and Cooper 2004; Hentges *et al.* 2006; Cavero *et al.* 2007). However, loss of *lig4* or *xlf1* gene causes dramatically enhanced IR sensitivity of *S. pombe* spores, which contain unreplicated genomes (Hentges *et al.* 2006). We hypothesized that if there are currently unknown *S. pombe* NHEJ factors, their loss should also result in a heightened level of IR sensitivity when cells are in the spore state, but not when cells are growing vegetatively. Thus, we decided to perform screens of the IR sensitivity phenotype using both vegetative cells and spores.

**Figure 1:**
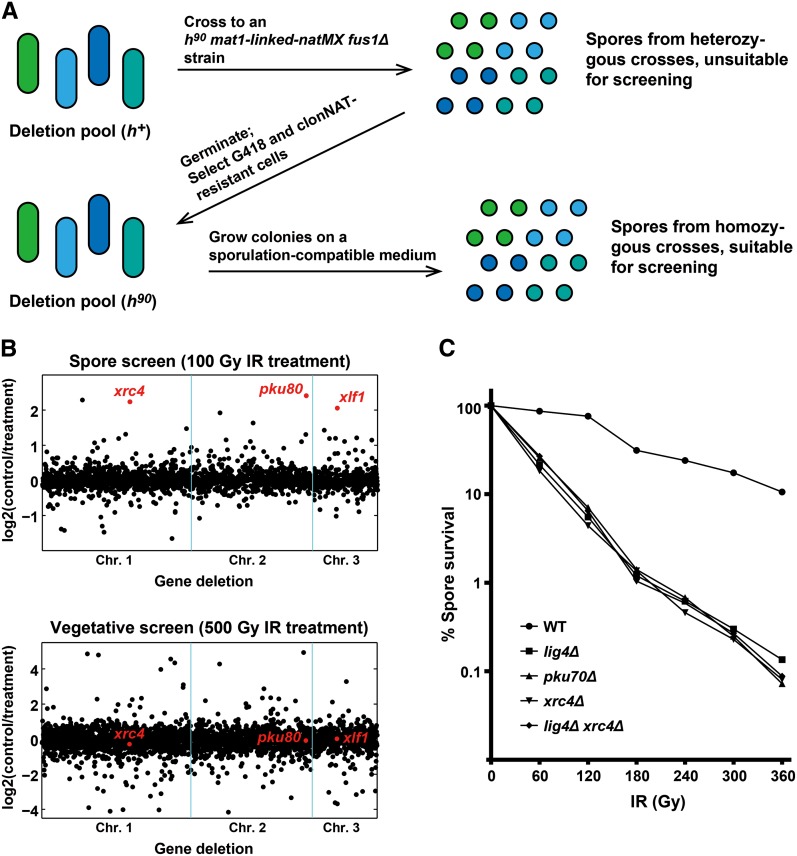
IR sensitivity screens identified *xrc4* as a gene required for IR resistance of spores. (A) The procedure used to generate a mutant spore pool for the spore IR sensitivity screen. (B) Scatter plots of the log2(control/treatment) ratios from the two IR sensitivity screens. Genes are ordered on the x-axis according to their chromosomal positions. *xrc4* and two known core NHEJ genes, *pku80* and *xlf1*, are highlighted in red. (C) Survival curves of spores treated with different doses of IR.

Previously, we have developed a deep-sequencing–based method for quantitatively phenotyping a fission yeast genome-wide haploid deletion collection (Han *et al.* 2010). This method takes advantage of the DNA barcodes in the deletion strains to track the abundance change of each strain in a mutant pool. It is straightforward to apply this method to the vegetative screen. However, to perform the spore screen, we could not simply generate spores by mating the deletion strains, whose mating type is *h^+^*, to an opposite mating type wild-type strain, because the protein products of wild-type genes will likely be present in the resulting spores regardless of their genotypes. Therefore, we devised a scheme to convert the deletion mutants to homothallic (self-mating) *h^90^* strains, so that spores can be derived from homozygous crosses (Figure 1A). In this scheme, we first mated the pooled deletion mutants with a specially constructed *h^90^* strain, DY4792, in which a *natMX* marker was inserted near the mating type locus *mat1*. DY4792 cells are *fus1^−^* so that they cannot mate with themselves but can mate with *fus1^+^* cells (Petersen *et al.* 1995). The progenies were selected using the antibiotics G418 and clonNAT, which enrich the *kanMX*-marked gene deletions from the mutant pool and the *natMX*-marked *h^90^* mating type, respectively. The enriched *h^90^* cells were then allowed to form single-clone colonies and produce spores through self-mating. The resulting spores were used for the IR sensitivity screen.

We chose the IR doses of 100 Gy and 500 Gy for the spore screen and the vegetative screen, respectively, because wild-type cells are more sensitive to IR in the spore state than in the vegetative state (J. Li *et al.*, unpublished observations). For each gene whose deletion is detectable by barcode sequencing, we calculated a normalized log2 ratio of sequencing read counts in untreated compared with IR-treated samples. For genes important for IR resistance, we expected a log2 ratio higher than 0 because the mutant cells should be depleted in the treated samples. We obtained log2 ratios for 2294 genes in the spore screen and 2859 genes in the vegetative screen (Figure 1B and Table S3). The lower number of scored genes in the spore screen is at least partially due to the loss of mating-defective mutants, which number in the hundreds (Sun *et al.* 2013), during the two rounds of mating needed for generating the spores.

We ranked the genes according to the log2 ratios and submitted the ranked lists to GSEA (Subramanian *et al.* 2005). As expected, among the genes whose mutants are IR-sensitive, DNA damage response genes are strongly enriched in both screens (FDR = 0.000 for the vegetative screen and FDR = 0.025 for the spore screen) (Figure S1).

There are four genes known to be essential for NHEJ in fission yeast: *pku70*, *pku80*, *lig4*, and *xlf1*. Consistent with the expectations, the deletion mutants of *pku80* and *xlf1* ranked among the most IR-sensitive mutants in the spore screen but did not show any IR sensitivity in the vegetative screen (Figure 1B and Table S3). There is no *lig4* mutant in the deletion collection we screened. The *pku70* mutant strain in this collection appeared to be problematic, because it did not show any IR sensitivity in the spore screen, whereas an independently made *pku70* deletion mutant displayed severe IR sensitivity in the spore state (Figure 1C). The deletion mutant of a previously uncharacterized gene, *SPAC6G9.16c*, behaved like the *pku80* and *xlf1* mutants in the screens, suggesting that this gene may also function in NHEJ. Because data shown below demonstrate that this gene encodes a homolog of human XRCC4, we named it *xrc4*. In this study, we focused on the characterization of *xrc4*, but our IR screen data should be a useful resource for future investigation of other genes.

### Xrc4 acts with Lig4 to promote the IR resistance of spores

To verify that Xrc4 is important for the IR resistance of spores, we constructed an *h^90^ xrc4Δ* strain by PCR-based gene targeting. Spores derived from this strain were significantly more sensitive to IR than wild-type spores (Figure 1C). The sensitivity of *xrc4Δ* spores was similar to that of *lig4Δ* spores and *pku70Δ* spores. Furthermore, *xrc4Δ lig4Δ* double mutant spores were no more sensitive than the single mutant spores, suggesting that Xrc4 and Lig4 act in the same pathway to promote IR resistance of spores.

### Xrc4 is a divergent homolog of human XRCC4 and budding yeast Lif1

**Figure 2:**
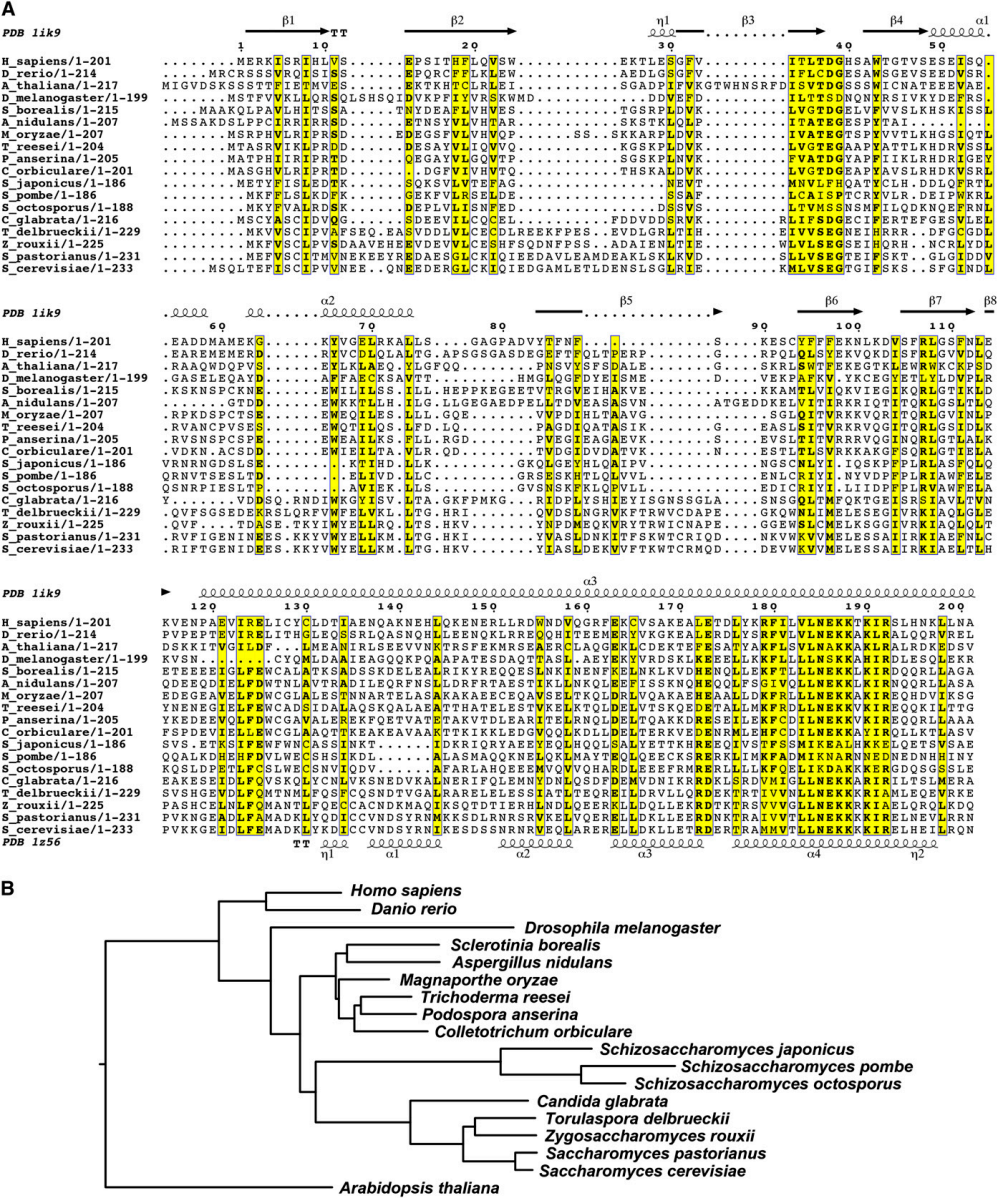
Fission yeast Xrc4 is a homolog of human XRCC4 and budding yeast Lif1. (A) Multiple sequence alignment of the N-terminal conserved region of XRCC4 family proteins. The alignment was generated using the MAFFT-L-INS-i method (http://mafft.cbrc.jp/alignment/server/) (Katoh and Standley 2013). Secondary structural elements of human XRCC4 (PDB 1ik9) and *S. cerevisiae* Lif1 (PDB 1z56) were visualized together with the sequence alignment using the ESPript 3.0 web server (http://espript.ibcp.fr/) (Gouet *et al.* 2003). (B) Phylogenetic tree based on the alignment in (A). The tree was constructed using the neighbor-joining (NJ) method (http://mafft.cbrc.jp/alignment/server/phylogeny.html) and visualized using FigTree (http://tree.bio.ed.ac.uk/software/figtree/). The *Arabidopsis* homolog of XRCC4 was used as the outgroup to root the tree. Protein sequence accession numbers are gi|12081905 (*Homo sapiens*), gi|37589745 (*Danio rerio*), gi|9800643 (*Arabidopsis thaliana*), gi|7294937 (*Drosophila melanogaster*), gi|563290357 (*Sclerotinia borealis*), gi|75858908 (*Aspergillus nidulans*), gi|389638394 (*Magnaporthe oryzae*), gi|572283599 (*Trichoderma reesei*), gi|171690284 (*Podospora anserina*), gi|477536394 (*Colletotrichum orbiculare*), and gi|530775004 (*Schizosaccharomyces japonicus*), gi|295443012 (*Schizosaccharomyces pombe*), gi|528062605 (*Schizosaccharomyces octosporus*), gi|27948821 (*Candida glabrata*), gi|367016485 (*Torulaspora delbrueckii*), gi|254585561 (*Zygosaccharomyces rouxii*), gi|113913533 (*Saccharomyces pastorianus*), and gi|6321348 (*Saccharomyces cerevisiae*).

Xrc4 is a protein of 264 amino acids. It is currently annotated as a “sequence orphan” by PomBase (Wood *et al.* 2012). We failed to uncover any obvious Xrc4 homologs outside of the *Schizosaccharomyces* genus by performing BLAST and PSI-BLAST searches. To boost the chance of detecting remote homologs, we turned to HHpred, a more sensitive homology search method that compares profile hidden Markov models (HMMs) (Söding 2005; Söding *et al.* 2005). A search against the HHpred pdb70 database using the HHpred web server led to >90% probability match between Xrc4 and human XRCC4 (PDB entry 1ik9), suggesting that Xrc4 is homologous to XRCC4. Multiple sequence alignment analysis lent support to the HHpred result (Figure 2A). The homology appears to span the N-terminal 186 amino acids of Xrc4, which correspond to the N-terminal 201 amino acids of human XRCC4. According to the crystal structures, this portion of XRCC4 contains its N-terminal globular head domain (amino acids 1–115) and central coiled-coil domain (amino acids 119–203) (Junop *et al.* 2000; Sibanda *et al.* 2001; Wu *et al.* 2009). Within this aligned region, the percentage identity between Xrc4 and human XRCC4 is 8.1%, and the percentage identity between Xrc4 and *S. cerevisiae* Lif1 is 9.3%. Such low levels of sequence identity explain why BLAST searches failed to reveal a connection between Xrc4 and XRCC4. Compared to many other fungal XRCC4 homologs, Xrc4 appears to have diverged farthest from the ancestral protein (Figure 2B).

### Xrc4 is required for DSB repair mediated by classical NHEJ

**Figure 3:**
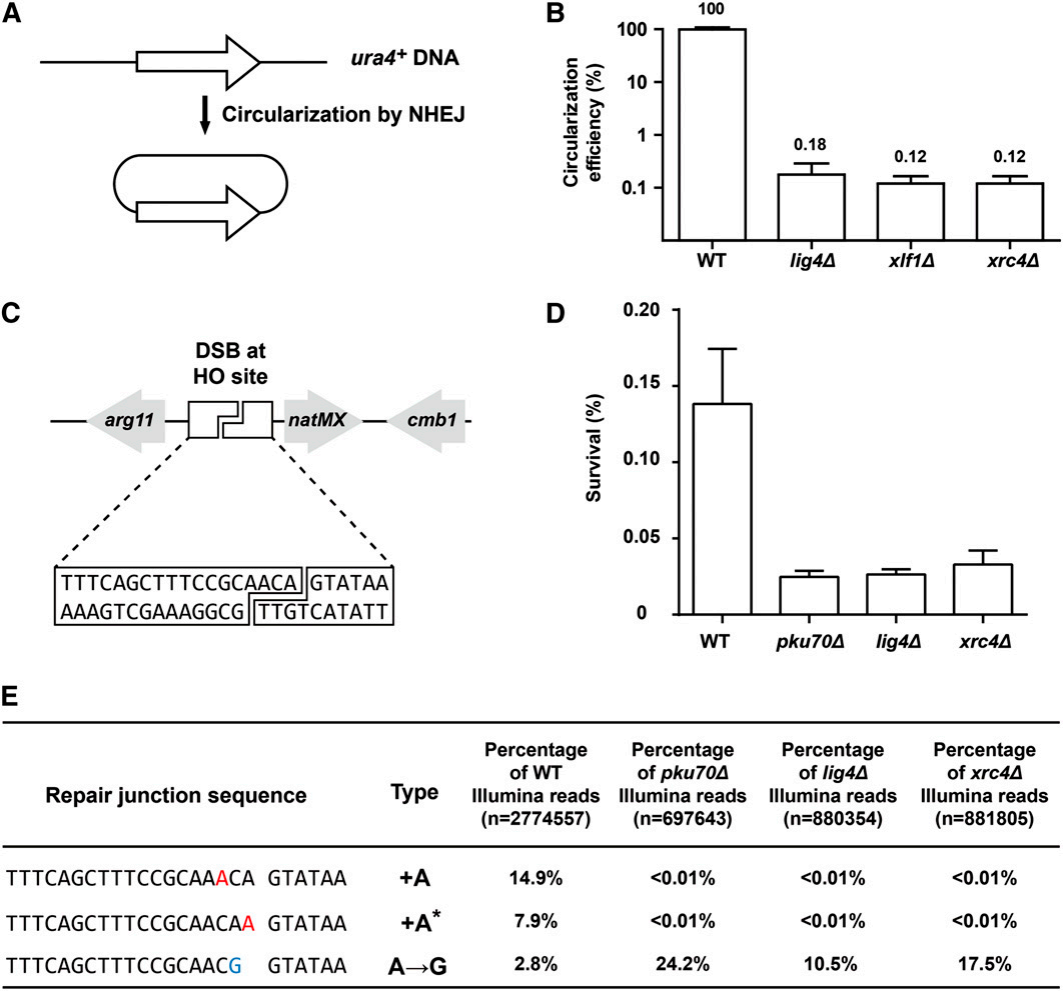
Xrc4 is required for classical NHEJ-mediated DSB repair. (A) Schematic of the *ura4^+^* circularization assay. (B) Like *lig4Δ* and *xlf1Δ*, *xrc4Δ* causes a severe defect in circularizing the linear *ura4^+^* DNA. The circularization efficiencies were normalized to that of the wild type. Error bars represent the SEM. (C) Schematic of the HO survivor assay. (D) Like *pku70Δ* and *lig4Δ*, *xrc4Δ* causes a reduction of HO survivor frequency. Error bars represent the SEM. (E) The HO repair junctions in *xrc4Δ* survivors share the same pattern as those in *pku70Δ* and *lig4Δ* survivors. The repair junction types are named as in Li *et al.* (2012). See Table S4 for all junctions with higher than 1% frequency in at least one of the four samples.

To determine whether Xrc4 participates in NHEJ, we performed two types of DSB repair assays. The first assay monitors an extrachromosomal DSB repair process, the circularization of a linear DNA fragment containing the *ura4^+^* gene (Figure 3A). As has been shown (Decottignies 2005), *ura4^+^* circularization is strongly dependent on NHEJ factors (Figure 3B). Compared to wild-type cells, the circularization efficiency decreased almost three orders of magnitude in *lig4Δ* and *xlf1Δ* cells. A similarly strong defect of *ura4^+^* circularization was observed for *xrc4Δ* cells, supporting the idea that Xrc4 acts in the same DSB repair process as Lig4 and Xlf1.

The second assay examines the imprecise end joining repair of a chromosomal DSB induced by the HO endonuclease (Figure 3C). This repair process deletes or mutates an HO cleavage site sequence to allow the cells to survive the continuous expression of HO. The imprecise repair events occurring in wild-type cells are mostly mediated by Pku70-dependent and Lig4-dependent classical NHEJ (Li *et al.* 2012). We found that, like *pku70Δ* and *lig4Δ*, deleting *xrc4* also caused a reduction of HO survival (Figure 3D). To directly examine the nature of the repair events, we performed deep sequencing analysis of the repair junctions in survivors of the wild-type, *pku70Δ*, *lig4Δ*, and *xrc4Δ* backgrounds (Figure 3E and Table S4). As previously found (Li *et al.* 2012), the two most frequent types of imprecise repair junctions in wild-type, the +A event and the +A* event, became virtually absent in *pku70Δ* and *lig4Δ*. However, a relatively rare event in the wild-type, the A→G event, became the most frequent imprecise repair event in *pku70Δ* and *lig4Δ*. In *xrc4Δ*, a repair junction pattern similar to those in *pku70Δ* and *lig4Δ* was observed. These results demonstrate that Xrc4 contributes to the imprecise repair of the HO-induced DSB in the same manner as Pku70 and Lig4.

### Lig4 and Xrc4 influence each other’s subcellular localization

**Figure 4:**
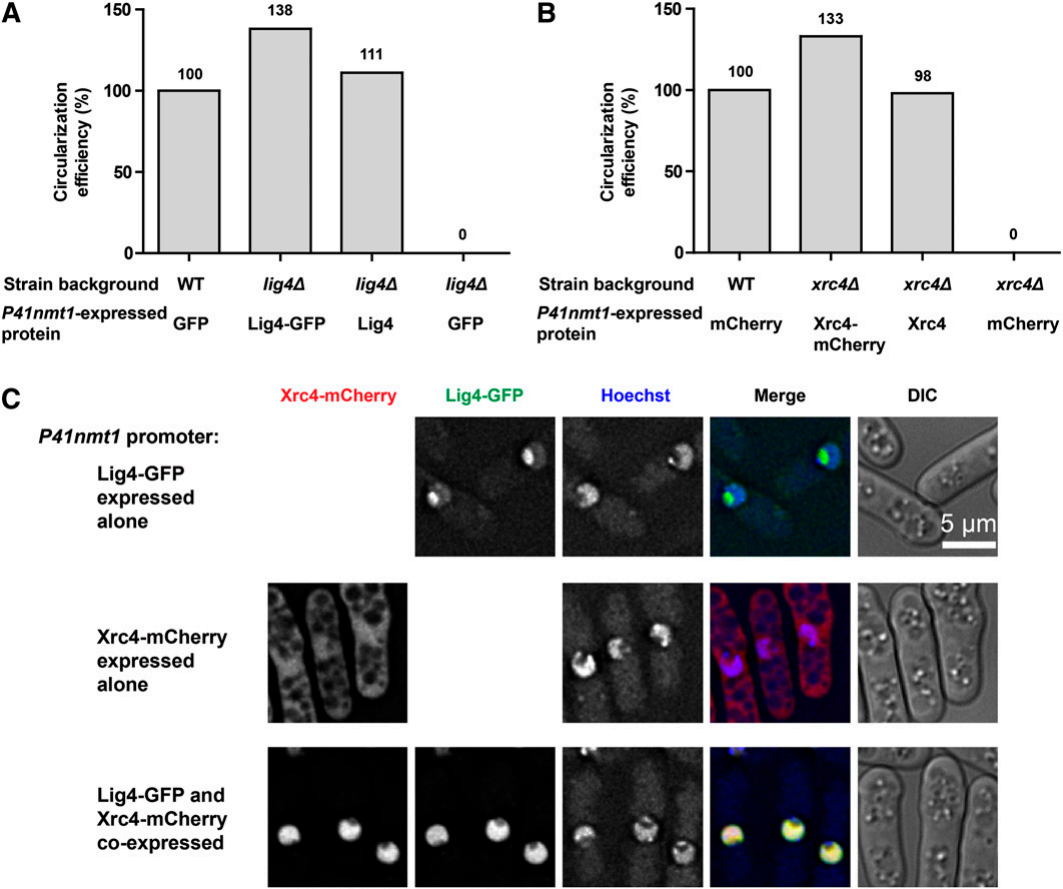
Xrc4 and Lig4 influence each other’s subcellular localization. (A) Lig4-GFP expressed from the *P41nmt1* promoter can rescue the *ura4^+^* circularization defect of *lig4Δ*. (B) Xrc4-mCherry expressed from the *P41nmt1* promoter can rescue the *ura4^+^* circularization defect of *xrc4Δ*. (C) The subcellular distribution of exogenously expressed Lig4-GFP and Xrc4-mCherry changed on co-expression. DNA was stained with Hoechst 33342.

Based on quantitative transcriptomics and proteomics data, both Lig4 and Xrc4 are expressed at a very low level (Marguerat *et al.* 2012). To facilitate the detection of these two proteins, we moderately overexpressed them using the attenuated *nmt1* promoter, *P41nmt1* (Basi *et al.* 1993). To visualize them by live cell imaging, we fused the green fluorescent protein GFP to the C-terminus of Lig4 and fused the red fluorescent protein mCherry to the C-terminus of Xrc4. Using the *ura4^+^* circularization assay, we found that both the untagged and tagged versions of Lig4 and Xrc4, when expressed from the *P41nmt1* promoter, fully complemented the DSB repair defect of the deletion mutants (Figure 4, A and B), indicating that neither the fluorescent protein fusion nor the exogenous promoter perturbed the functions of Lig4 and Xrc4.

When Xrc4-mCherry was expressed alone in the wild-type cells using the *P41nmt1* promoter, it distributed evenly in the cytoplasm and nucleus (Figure 4C). When Lig4-GFP was expressed alone in the wild-type cells using the *P41nmt1* promoter, it mainly localized inside the nucleus, with a higher concentration in the nucleolus, the portion of the nucleus not stained by the DNA binding dye Hoechst 33342 (Figure 4C). Interestingly, when Xrc4-mCherry and Lig4-GFP were expressed together, they both concentrated in the DNA dye–stained region of the nucleus, which is termed the nuclear chromatin region in fission yeast (Toda *et al.* 1981). Thus, these two proteins regulate each other’s nuclear localization.

### Xrc4 physically interacts with Lig4

**Figure 5:**
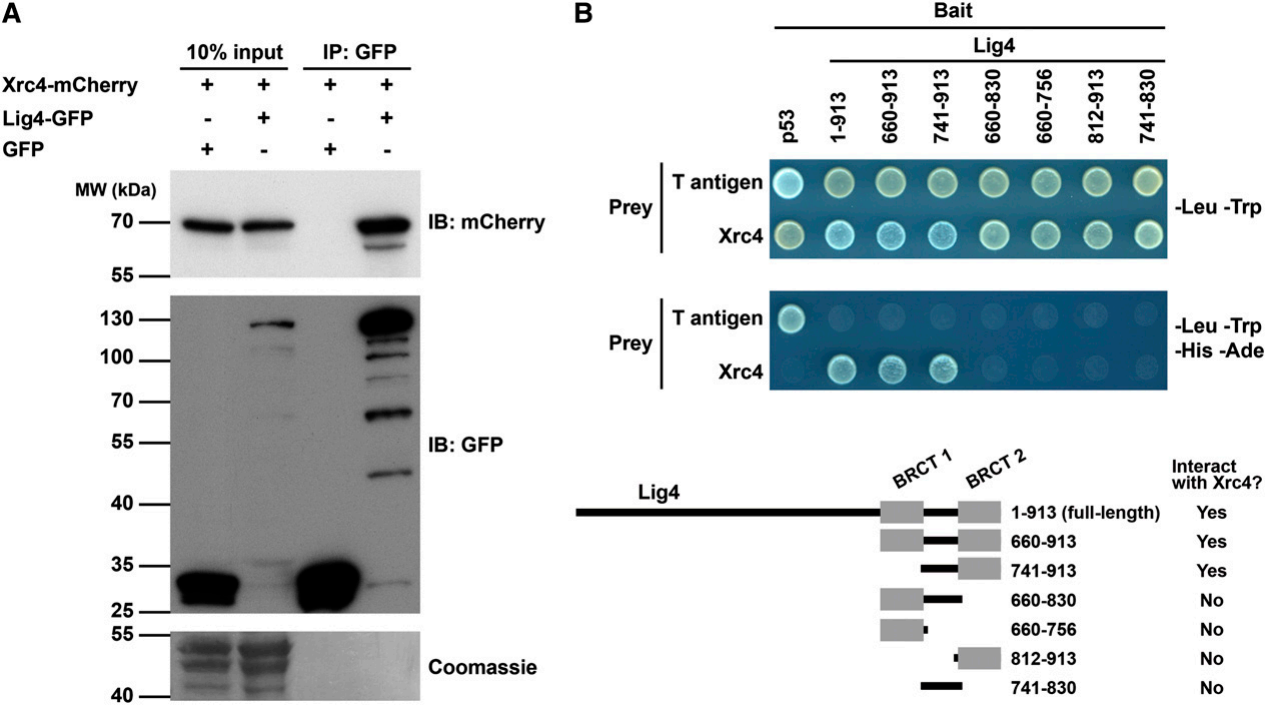
Xrc4 and Lig4 physically interact with each other. (A) Xrc4-mCherry can be co-immunoprecipitated with Lig4-GFP. Coomassie staining of PVDF membrane after immunodetection was used to control for protein loading and blotting efficiency (Welinder and Ekblad 2011). (B) Xrc4 and Lig4 interact in the yeast two-hybrid assay and the interaction requires the inter-BRCT linker and the BRCT2 domain of Lig4.

To determine whether Xrc4 physically interacts with Lig4, we performed co-immunoprecipitation analysis (Figure 5A). When Lig4-GFP and Xrc4-mCherry were co-expressed, Xrc4-mCherry was co-immunoprecipitated with Lig4-GFP. As a control, when GFP and Xrc4-mCherry were co-expressed, Xrc4-mCherry was not co-immunoprecipitated with GFP. Thus, Lig4 and Xrc4 associate with each other specifically.

To examine whether Xrc4 and Lig4 can engage in a binary interaction in the absence of other *S. pombe* proteins, we used the yeast two-hybrid assay (Figure 5B). Lig4 and Xrc4 exhibited a strong two-hybrid interaction with each other, but not with the control proteins T antigen and p53, respectively. Like the interaction between human XRCC4 and LigIV (Critchlow *et al.* 1997), and the interaction between *S. cerevisiae* Lif1 and Dnl4 (Herrmann *et al.* 1998), a C-terminal fragment of Lig4 (amino acids 660–913), which contains two BRCT domains, is sufficient for interacting with Xrc4. Further truncation analysis showed that either BRCT domain alone or the linker region between the two BRCT domains is not capable of interacting with Xrc4. The minimal Lig4 fragment that can support this interaction (amino acids 741–913) encompasses both the inter-BRCT linker and the BRCT2 domain. In contrast, the fragment encompassing the BRCT1 domain and the linker (amino acids 660–830) failed to interact with Xrc4. These results are consistent with previous observations that for budding yeast Dnl4 and human LigIV, the inter-BRCT linker and the BRCT2 domain, but not the BRCT1 domain, are important for the interactions with Lif1 and XRCC4, respectively (Herrmann *et al.* 1998; Wu *et al.* 2009).

## Discussion

In this study, we found a new fission yeast NHEJ factor Xrc4 through genome-wide screens for radiosensitive mutants. Multiple lines of evidence demonstrate that Xrc4 acts together with Lig4 and is an ortholog of human XRCC4. First, HHpred searches revealed a sequence homology between Xrc4 and XRCC4. Second, the *xrc4* mutant exhibits the same spore IR sensitivity phenotype as the *lig4* mutant, and the double mutant is no more sensitive than the single mutants. Third, the *xrc4* mutant shows the same defect in DSB repair assays as the *lig4* mutant. Fourth, Xrc4 and Lig4 influence each other’s subcellular localization. Finally, Xrc4 physically interacts with Lig4 through a Lig4 region homologous to the XRCC4-binding region of human LigIV.

### Evolutionary divergence of XRCC4 family proteins

It has been noted that the sequence divergence between human XRCC4 and budding yeast Lif1 is surprisingly large given their functional conservation (Herrmann *et al.* 1998; Grawunder *et al.* 1998a). The discovery of fission yeast Xrc4 indicates that the level of divergence within this protein family is even higher than previously appreciated. Why is there so much divergence among XRCC4 family proteins? Based on observations of highly divergent orthologs between *S. pombe* and *S. cerevisiae*, Wood has suggested that these proteins are often part of protein complexes and do not interact directly with invariable molecules (*e.g.*, ATP), and thus can freely evolve as long as the protein–protein interaction interface is maintained by compensatory changes (Wood 2006). XRCC4 proteins appear to conform to such a pattern, and we note that the unusually large divergence of *S. pombe* Xrc4 is accompanied by a lack of conservation in the inter-BRCT linker region of *S. pombe* Lig4 (Figure S2), suggesting that the interaction interface between Xrc4 and Lig4 may have undergone large but compensatory changes.

Genes encoding NHEJ factors in *Saccharomyces* yeasts and primates, including the *XRCC4* genes in primates, have been found to undergo accelerated evolution, perhaps due to the involvement of the NHEJ pathway in genome defense against transposons and viruses (Sawyer and Malik 2006; Demogines *et al.* 2010). The selective force exerted on the NHEJ factors by an evolutionary arms race may be another reason why XRCC4 family proteins have diverged so much.

Among the yeast species belonging to the Saccharomycetaceae family, *Candida albicans* and *Ashbya gossypii* lack a recognizable homolog of XRCC4 but do have apparent homologs of Ku, LigIV, and XLF (Wilson 2007). It is possible that XRCC4 homologs in these species have diverged beyond recognition like fission yeast Xrc4. An alternative explanation is gene degeneration or loss, as has been shown for another Saccharomycetaceae species, *Lachancea kluyveri*, in which genes encoding the homologs of LigIV, XRCC4, and XLF have all been pseudogenized or lost (Gordon *et al.* 2011). To our knowledge, there is no experimental evidence supporting the existence of a functional NHEJ pathway in *C. albicans* or *A. gossypii*.

Some of the explanations we offer for the divergence of XRCC4 family proteins may also apply to the XLF/Nej1 family proteins, which display an equally notable lack of conservation (Callebaut *et al.* 2006; Hentges *et al.* 2006; Wilson 2007; Cavero *et al.* 2007; Deshpande and Wilson 2007).

### The roles of Xrc4 in NHEJ

As part of a ligase complex, human XRCC4 and budding yeast Lif1 promote the ligation reaction in multiple ways, which include: stabilizing the catalytic subunit of the ligase complex (Herrmann *et al.* 1998; Bryans *et al.* 1999); stimulating the enzymatic activities of the catalytic subunit (Grawunder *et al.* 1997; Teo and Jackson 2000); targeting the catalytic subunit to DSBs (Teo and Jackson 2000; Mari *et al.* 2006); and promoting the nuclear import and proper sub-nuclear distribution of the catalytic subunit (Berg *et al.* 2011). Fission yeast Xrc4 is expected to share at least some of these functions. Our live imaging analysis of exogenously expressed Xrc4 and Lig4 suggests that they regulate each other’s nuclear localizations. We have searched for nuclear localization signals (NLS) in Xrc4 and Lig4 using computational tools. Two different software, cNLS Mapper (Kosugi *et al.* 2009) and NLStradamus (Nguyen Ba *et al.* 2009), predicted the presence of an NLS within the linker region (amino acids 630–659) between the catalytic domain and the first BRCT domain of Lig4. This is exactly the same location where a bipartite NLS (amino acids 623–638) was experimentally defined in human LigIV (Girard *et al.* 2004). In contrast, neither of the software was able to find an NLS in Xrc4. The experimentally identified NLS of XRCC4 is located at its C-terminal tail region (Grawunder *et al.* 1998b; Girard *et al.* 2004), which is not conserved in the fungal XRCC4 homologs. Thus, the dependence of Xrc4 nuclear localization on co-expressed Lig4 is likely due to the absence of an NLS in Xrc4. Interestingly, despite its ability to localize to the nucleus when expressed alone, Lig4 needs co-expressed Xrc4 to concentrate at the chromatin region, suggesting that the ligase complex has a higher affinity for DNA or certain DNA-bound proteins than Lig4 alone. This is similar to the situation in human cells, where co-expression of XRCC4 led to the exclusion of LigIV from nucleoli (Berg *et al.* 2011). Thus, fission yeast Xrc4 and human XRCC4 may possess a similar ability to promote the association of the ligase complex with chromatin.

In addition to interacting with the catalytic subunit of the ligase complex, human XRCC4 and budding yeast Lif1 also serves as protein-interaction hubs for bringing the ligase complex into contact with many other NHEJ factors, which include XLF/Nej1 (Frank-Vaillant and Marcand 2001; Palmbos *et al.* 2005; Deshpande and Wilson 2007), the Mre11-Rad50-Xrs2/Nbs1 (MRX/MRN) complex (Palmbos *et al.* 2005, 2008; Matsuzaki *et al.* 2008), Ku70/80 heterodimer (Mari *et al.* 2006), polynucleotide kinase (PNK) (Koch *et al.* 2004), aprataxin (Clements *et al.* 2004), and PNK-like and aprataxin-like factor (PALF) (Kanno *et al.* 2007; Iles *et al.* 2007; Macrae *et al.* 2008). Fission yeast Xrc4 may engage in similar interactions. For example, its Thr261 residue lies in a sequence motif (SDTVSE) that matches both the CK2 phosphorylation site consensus and the Nbs1 FHA-binding consensus (Lloyd *et al.* 2009; Williams *et al.* 2009), and thus may mediate a phosphorylation-dependent interaction with the FHA domain of Nbs1, similar to the interaction between budding yeast Lif1 and the FHA domain of Xrs2 (Palmbos *et al.* 2008).

The functions of human XRCC4 and budding yeast Lif1 are subject to regulation by post-translational modifications, which include phosphorylation by DNA-PK (Yu *et al.* 2003), CK2 (Koch *et al.* 2004), and cyclin-dependent kinase (CDK) (Matsuzaki *et al.* 2012), and SUMOylation (Yurchenko *et al.* 2006; Vigasova *et al.* 2013). It will be interesting to know whether fission yeast Xrc4 is also regulated by such means.

NHEJ activity is reported to be 10-times higher in the G1 than in the G2 phase of the cell cycle in fission yeast, but the underlying mechanism remains unknown (Ferreira and Cooper 2004). The identification of Xrc4 will help future efforts to address this and other interesting aspects of NHEJ regulation in fission yeast.

## Supplementary Material

Supporting Information
