# Supplementary material for: Genome-wide Screens for Sensitivity to Ionizing Radiation Identify the Fission Yeast Nonhomologous End Joining Factor Xrc4
Source: G3 (Bethesda). 2014 May 21;4(7):1297–306. doi: 10.1534/g3.114.011841 (PMC4455778; doi:10.1534/g3.114.011841)
Supplement: Supporting Information [file supp_4_7_1297__index.html]

Genome-wide Screens for Sensitivity to Ionizing Radiation Identify the Fission Yeast Nonhomologous End Joining Factor Xrc4 — Supporting Information 

# Genome-wide Screens for Sensitivity to Ionizing Radiation Identify the Fission Yeast Nonhomologous End Joining Factor Xrc4

## Supporting Information for Li *et al.*, 2014

**Files in this Data Supplement:**

- Supporting Information - Figures S1-S2 and Tables S1-S4 (PDF, 582 KB)
- Figure S1 - GSEA enrichment plots for the gene set RESPONSE\_TO\_DNA\_DAMAGE\_STIMULUS. (PDF, 474 KB)
- Figure S2 - A sequence alignment of the C-terminal regions of human LigIV, fission yeast Lig4, and budding yeast Dnl4. (PDF, 400 KB)
- Table S1 - Fission yeast strains used in this study. (PDF, 117 KB)
- Table S2 - Plasmids used in this study. (PDF, 115 KB)
- Table S3 - The log2(control/treatment) ratios of the Bioneer deletion mutants in the vegetative and spore screens. (.xls, 639 KB)
- Table S4 - The HO repair junctions revealed by deep sequencing. (.xls, 30 KB)
