# Supplementary material for: Genome-wide Screens for Sensitivity to Ionizing Radiation Identify the Fission Yeast Nonhomologous End Joining Factor Xrc4
Source: G3 (Bethesda). 2014 May 21;4(7):1297–306. doi: 10.1534/g3.114.011841 (PMC4455778; doi:10.1534/g3.114.011841)
Supplement: Supporting Information [file supp_g3.114.011841_011841SI.pdf]

G3: Genes | Genomes | Genetics

Genome-wide screens for sensitivity to ionizing radiation identify the fission yeast nonhomologous end joining factor Xrc4

Jun Li[1], Yang Yu[1], Fang Suo, Ling-Ling Sun, Dan Zhao, Li-Lin Du
National Institute of Biological Sciences, Beijing 102206, China
[1]These authors contributed equally to this work.

Correspondence should be addressed to L.-L. D.
National Institute of Biological Sciences, 7 Science Park Road, Zhongguancun Life Science Park, Beijing 102206, China
e-mail: dulilin@nibs.ac.cn

DOI: 10.1534/g3.114.011841

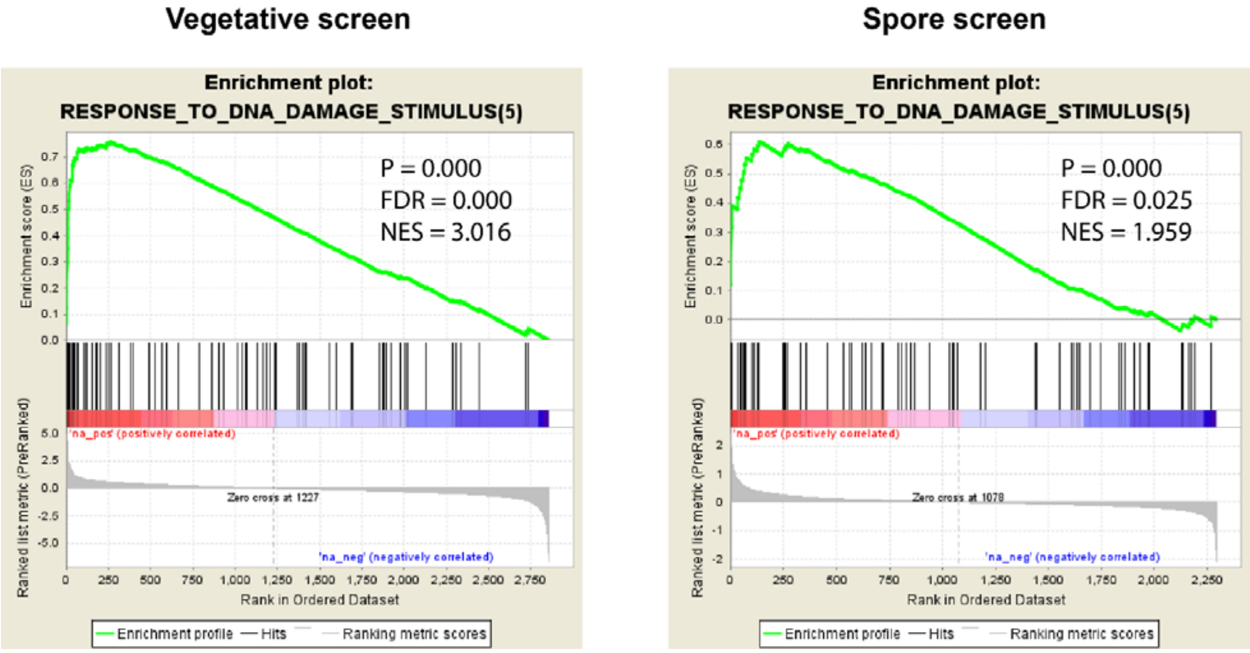

Figure S1 GSEA enrichment plots for the gene set RESPONSE_TO_DNA_DAMAGE_STIMULUS.

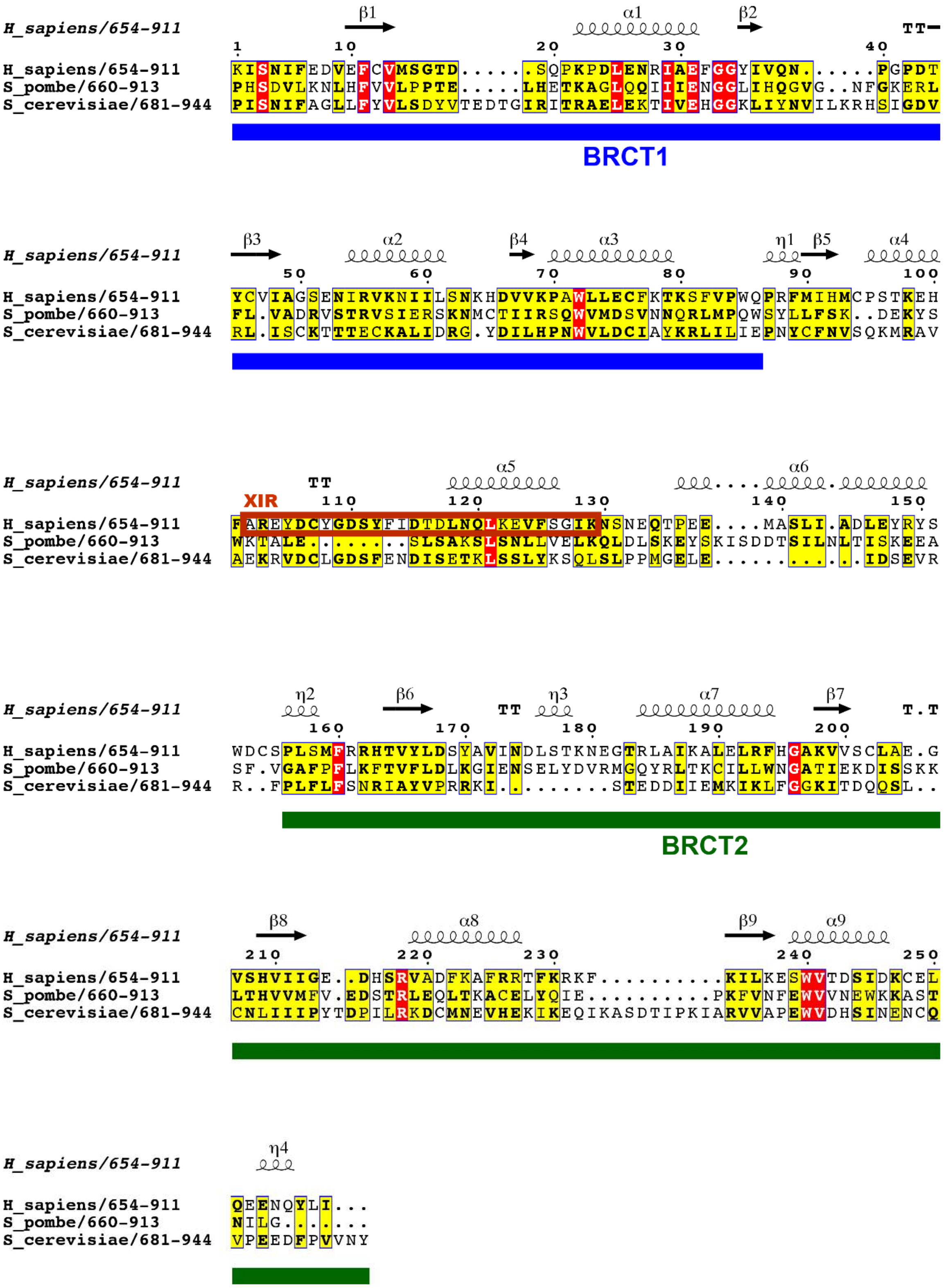

Figure S2 A sequence alignment of the C-terminal regions of human LigIV, fission yeast Lig4, and budding yeast Dnl4. The alignment was generated using the MAFFT-L-INS-i method. Secondary structural elements of human LigIV (PDB 3ii6, chain X) was visualized together with the sequence alignment using the ESPript 3.0 web server (http://espript.ibcp.fr/) (Gouet *et al.* 2003). Blue bar denotes the BRCT1 domain, green bar denotes the BRCT2 domain, and the red rectangle denotes a 28-amino-acid sequence termed "XRCC4-interacting region" (XIR) (amino acids 755-782) within the inter-BRCT linker of human LigIV (Sibanda *et al.* 2001).

Table S1 Fission yeast strains used in this study

Strain	Genotype
DY4792	h^{90} *leu1-32 ura4-D18 ade6-216 fus1Δ::hphMX mat1-linked-natMX*
DY3207	h^{90} *leu1-32*
DY4442	h^{90} *leu1-32 pku70Δ::kanMX*
DY4428	h^{90} *leu1-32 lig4Δ::kanMX*
DY16147	h^{90} *leu1-32 xrc4Δ::natMX*
DY16149	h^{90} *leu1-32 lig4Δ::kanMX xrc4Δ::natMX*
LD260	h^{-} *ura4-D18 his3-D1 leu1-32*
DY8501	h^{-} *ura4-D18 his3-D1 leu1-32 lig4Δ::kanMX*
DY15887	h^{-} *ura4-D18 his3-D1 leu1-32 xlf1Δ::natMX*
DY8497	h^{-} *ura4-D18 his3-D1 leu1-32 xrc4Δ::kanMX*
DY49	h^{+} *leu1-32 his3-D1 arg3Δ::HOsite-natMX ars1::[pJR1-41XH+HO](his3*$^{+}$*)*
DY2876	h^{+} *leu1-32 his3-D1 arg3Δ::HOsite-natMX ars1::[pJR1-41XH+HO](his3*$^{+}$*) pku70Δ::kanMX*
DY2879	h^{+} *leu1-32 his3-D1 arg3Δ::HOsite-natMX ars1::[pJR1-41XH+HO](his3*$^{+}$*) lig4Δ::kanMX*
DY6657	h^{-} *leu1-32 his3-D1 arg3Δ::HOsite-natMX ars1::[pJR1-41XH+HO](his3*$^{+}$*) xrc4Δ::kanMX*
DY15895	h^{-} *ura4-D18 his3-D1 leu1-32::P41nmt1-GFP(leu1*$^{+}$*)*
DY15896	h^{-} *ura4-D18 his3-D1 leu1-32::P41nmt1-lig4-GFP(leu1*$^{+}$*) lig4Δ::kanMX*
DY15897	h^{-} *ura4-D18 his3-D1 leu1-32::P41nmt1-lig4(leu1*$^{+}$*) lig4Δ::kanMX*
DY15898	h^{-} *ura4-D18 his3-D1 leu1-32::P41nmt1-GFP(leu1*$^{+}$*) lig4Δ::kanMX*
DY15891	h^{-} *ura4-D18 his3-D1 leu1-32::P41nmt1-mCherry(leu1*$^{+}$*)*
DY15892	h^{-} *ura4-D18 his3-D1 leu1-32::P41nmt1-xrc4-mCherry(leu1*$^{+}$*) xrc4Δ::kanMX*
DY15893	h^{-} *ura4-D18 his3-D1 leu1-32::P41nmt1-xrc4(leu1*$^{+}$*) xrc4Δ::kanMX*
DY15894	h^{-} *ura4-D18 his3-D1 leu1-32::P41nmt1-mCherry(leu1*$^{+}$*) xrc4Δ::kanMX*
DY15901	h^{-} *ura4-D18 his3-D1 leu1-32::P41nmt1-lig4-GFP(leu1*$^{+}$*)*
DY15886	h^{-} *ura4-D18 his3-D1 leu1-32::P41nmt1-xrc4-mCherry:: P41nmt1-GFP(SVEM-hph)*
DY15885	h^{-} *ura4-D18 his3-D1 leu1-32::P41nmt1-xrc4-mCherry:: P41nmt1-lig4-GFP(SVEM-hph)*

Table S2 Plasmids used in this study

Plasmid	Description
pDB2673	pDUAL+P41nmt1-lig4-GFP
pDB2675	pDUAL+P41nmt1-lig4
pDB2679	pDUAL+P41nmt1-xrc4-mCherry
pDB2680	pDUAL+P41nmt1-xrc4
pDB1751	pDUAL+P41nmt1-GFP
pDB1626	pDUAL+P41nmt1-mCherry
pDB2144	pJK148+SVEM-hph
pDB2620	Y2H-bait-vector+p53
pDB2621	Y2H-prey-vector+T-antigen
pDB2289	Y2H-bait-vector+lig4(1-913)
pDB2622	Y2H-bait-vector+lig4(660-913)
pDB2624	Y2H-bait-vector+lig4(741-913)
pDB2623	Y2H-bait-vector+lig4(660-830)
pDB2625	Y2H-bait-vector+lig4(660-756)
pDB2626	Y2H-bait-vector+lig4(812-913)
pDB2627	Y2H-bait-vector+lig4(741-830)
pDB2288	Y2H-prey-vector+xrc4

Tables S3-S4
Available for download as Excel files at http://www.g3journal.org/lookup/suppl/doi:10.1534/g3.114.011841/-/DC1

Table S3 The log2(control/treatment) ratios of the Bioneer deletion mutants in the vegetative and spore screens.

Table S4 The HO repair junctions revealed by deep sequencing. The junctions with higher than 1% frequency in at least one of the four samples are shown.
