# Supplementary material for: Genome-wide Screens for Sensitivity to Ionizing Radiation Identify the Fission Yeast Nonhomologous End Joining Factor Xrc4
Source: G3 (Bethesda). 2014 May 21;4(7):1297–306. doi: 10.1534/g3.114.011841 (PMC4455778; doi:10.1534/g3.114.011841)
Supplement: Supporting Information [file supp_g3.114.011841_FigureS1.pdf]

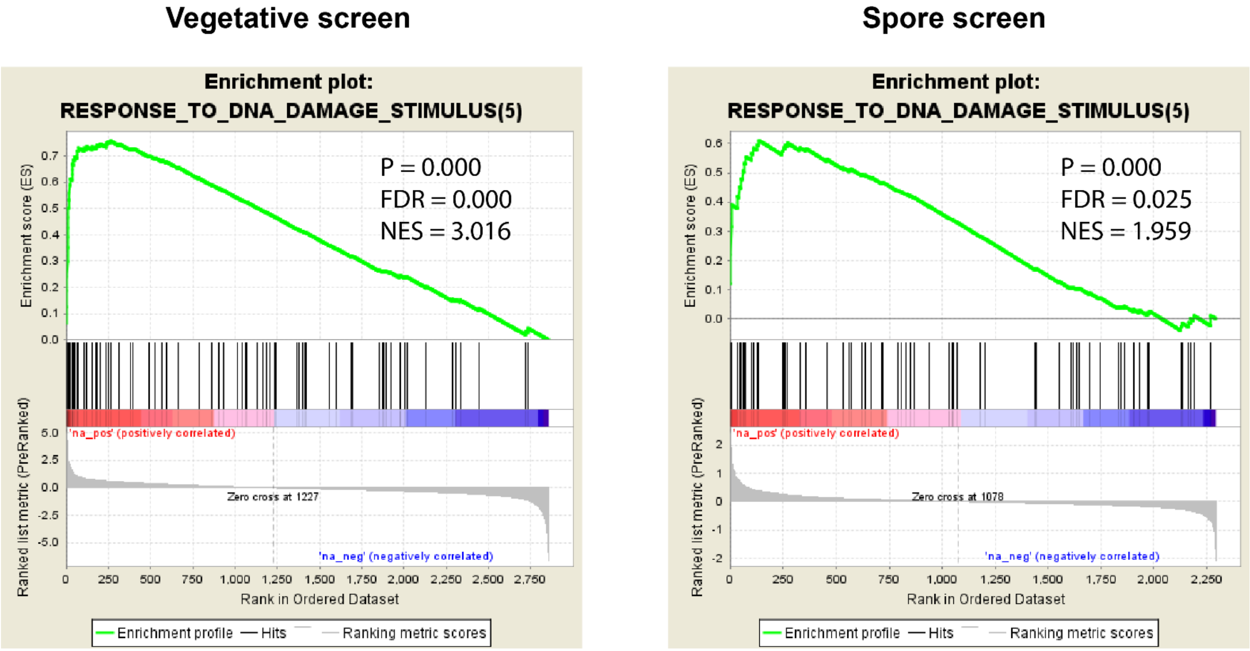

Figure S1 GSEA enrichment plots for the gene set RESPONSE_TO_DNA_DAMAGE_STIMULUS.
