# Supplementary material for: Genome-wide Screens for Sensitivity to Ionizing Radiation Identify the Fission Yeast Nonhomologous End Joining Factor Xrc4
Source: G3 (Bethesda). 2014 May 21;4(7):1297–306. doi: 10.1534/g3.114.011841 (PMC4455778; doi:10.1534/g3.114.011841)
Supplement: Supporting Information [file supp_g3.114.011841_TableS1.pdf]

Table S1 Fission yeast strains used in this study

Strain	Genotype
DY4792	*h^{90} leu1-32 ura4-D18 ade6-216 fus1Δ::hphMX mat1-linked-natMX*
DY3207	*h^{90} leu1-32*
DY4442	*h^{90} leu1-32 pku70Δ::kanMX*
DY4428	*h^{90} leu1-32 lig4Δ::kanMX*
DY16147	*h^{90} leu1-32 xrc4Δ::natMX*
DY16149	*h^{90} leu1-32 lig4Δ::kanMX xrc4Δ::natMX*
LD260	*h^{-} ura4-D18 his3-D1 leu1-32*
DY8501	*h^{-} ura4-D18 his3-D1 leu1-32 lig4Δ::kanMX*
DY15887	*h^{-} ura4-D18 his3-D1 leu1-32 xlf1Δ::natMX*
DY8497	*h^{-} ura4-D18 his3-D1 leu1-32 xrc4Δ::kanMX*
DY49	*h^{+} leu1-32 his3-D1 arg3Δ::HOsite-natMX ars1::[pJR1-41XH+HO](his3^{+})*
DY2876	*h^{+} leu1-32 his3-D1 arg3Δ::HOsite-natMX ars1::[pJR1-41XH+HO](his3^{+}) pku70Δ::kanMX*
DY2879	*h^{+} leu1-32 his3-D1 arg3Δ::HOsite-natMX ars1::[pJR1-41XH+HO](his3^{+}) lig4Δ::kanMX*
DY6657	*h^{-} leu1-32 his3-D1 arg3Δ::HOsite-natMX ars1::[pJR1-41XH+HO](his3^{+}) xrc4Δ::kanMX*
DY15895	*h^{-} ura4-D18 his3-D1 leu1-32::P41nmt1-GFP(leu1^{+})*
DY15896	*h^{-} ura4-D18 his3-D1 leu1-32::P41nmt1-lig4-GFP(leu1^{+}) lig4Δ::kanMX*
DY15897	*h^{-} ura4-D18 his3-D1 leu1-32::P41nmt1-lig4(leu1^{+}) lig4Δ::kanMX*
DY15898	*h^{-} ura4-D18 his3-D1 leu1-32::P41nmt1-GFP(leu1^{+}) lig4Δ::kanMX*
DY15891	*h^{-} ura4-D18 his3-D1 leu1-32::P41nmt1-mCherry(leu1^{+})*
DY15892	*h^{-} ura4-D18 his3-D1 leu1-32::P41nmt1-xrc4-mCherry(leu1^{+}) xrc4Δ::kanMX*
DY15893	*h^{-} ura4-D18 his3-D1 leu1-32::P41nmt1-xrc4(leu1^{+}) xrc4Δ::kanMX*
DY15894	*h^{-} ura4-D18 his3-D1 leu1-32::P41nmt1-mCherry(leu1^{+}) xrc4Δ::kanMX*
DY15901	*h^{-} ura4-D18 his3-D1 leu1-32::P41nmt1-lig4-GFP(leu1^{+})*
DY15886	*h^{-} ura4-D18 his3-D1 leu1-32::P41nmt1-xrc4-mCherry:: P41nmt1-GFP(SVEM-hph)*
DY15885	*h^{-} ura4-D18 his3-D1 leu1-32::P41nmt1-xrc4-mCherry:: P41nmt1-lig4-GFP(SVEM-hph)*
