# Supplementary material for: Genome-wide Screens for Sensitivity to Ionizing Radiation Identify the Fission Yeast Nonhomologous End Joining Factor Xrc4
Source: G3 (Bethesda). 2014 May 21;4(7):1297–306. doi: 10.1534/g3.114.011841 (PMC4455778; doi:10.1534/g3.114.011841)
Supplement: Supporting Information [file supp_g3.114.011841_TableS2.pdf]

Table S2 Plasmids used in this study

Plasmid	Description
pDB2673	pDUAL+P41nmt1-lig4-GFP
pDB2675	pDUAL+P41nmt1-lig4
pDB2679	pDUAL+P41nmt1-xrc4-mCherry
pDB2680	pDUAL+P41nmt1-xrc4
pDB1751	pDUAL+P41nmt1-GFP
pDB1626	pDUAL+P41nmt1-mCherry
pDB2144	pJK148+SVEM-hph
pDB2620	Y2H-bait-vector+p53
pDB2621	Y2H-prey-vector+T-antigen
pDB2289	Y2H-bait-vector+lig4(1-913)
pDB2622	Y2H-bait-vector+lig4(660-913)
pDB2624	Y2H-bait-vector+lig4(741-913)
pDB2623	Y2H-bait-vector+lig4(660-830)
pDB2625	Y2H-bait-vector+lig4(660-756)
pDB2626	Y2H-bait-vector+lig4(812-913)
pDB2627	Y2H-bait-vector+lig4(741-830)
pDB2288	Y2H-prey-vector+xrc4
