# Supplementary material for: Genome-wide Screens for Sensitivity to Ionizing Radiation Identify the Fission Yeast Nonhomologous End Joining Factor Xrc4
Source: G3 (Bethesda). 2014 May 21;4(7):1297–306. doi: 10.1534/g3.114.011841 (PMC4455778; doi:10.1534/g3.114.011841)
Supplement: Supporting Information [file supp_g3.114.011841_FigureS2.pdf]

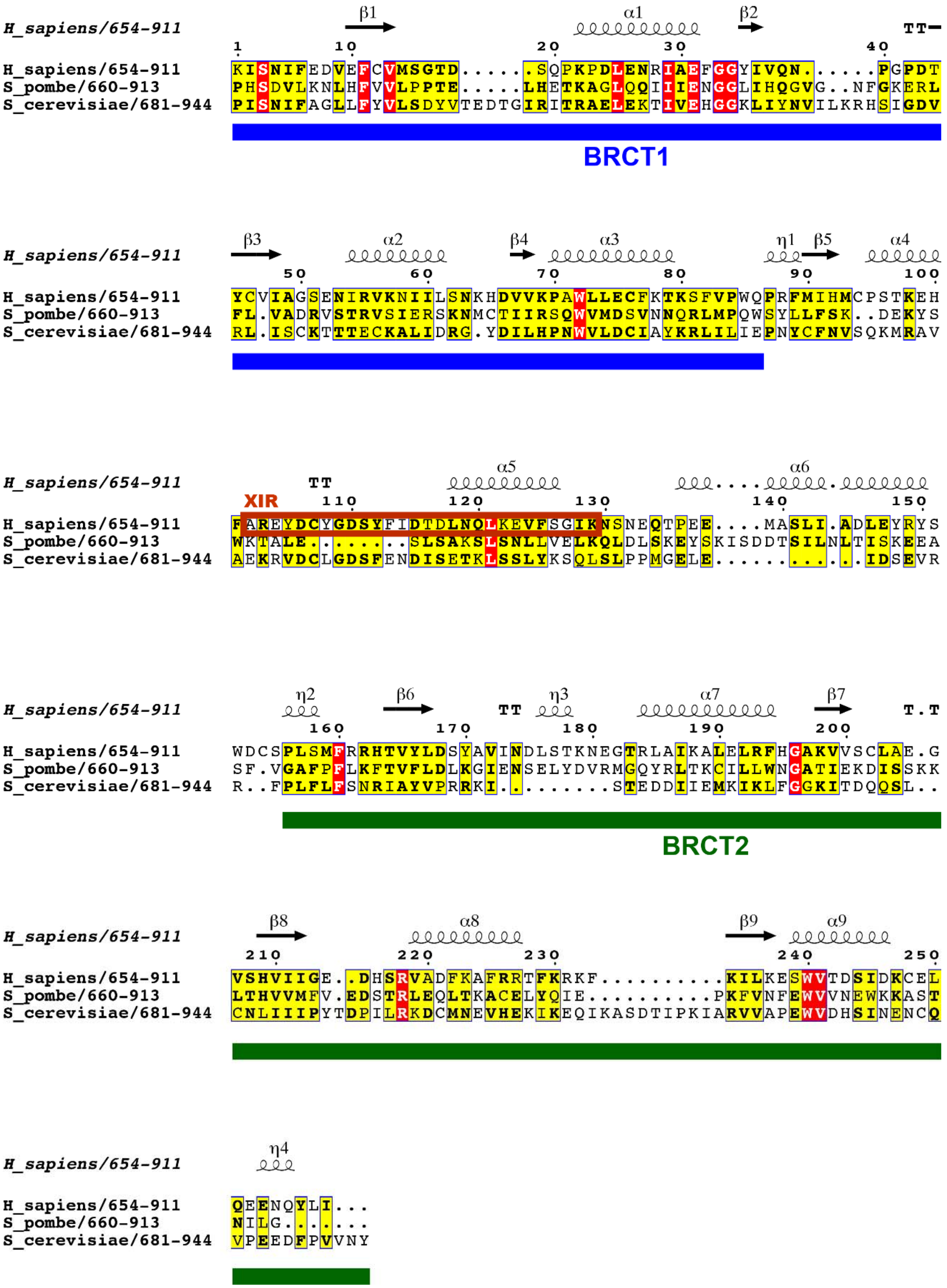

Figure S2 A sequence alignment of the C-terminal regions of human LigIV, fission yeast Lig4, and budding yeast Dnl4. The alignment was generated using the MAFFT-L-INS-i method. Secondary structural elements of human LigIV (PDB 3ii6, chain X) was visualized together with the sequence alignment using the ESPript 3.0 web server (http://espript.ibcp.fr/) (Gouet *et al.* 2003). Blue bar denotes the BRCT1 domain, green bar denotes the BRCT2 domain, and the red rectangle denotes a 28-amino-acid sequence termed "XRCC4-interacting region" (XIR) (amino acids 755-782) within the inter-BRCT linker of human LigIV (Sibanda *et al.* 2001).
